# Structure Determination of Microtubules and Pili: Past, Present, and Future Directions

**DOI:** 10.3389/fmolb.2021.830304

**Published:** 2022-01-14

**Authors:** James A. Garnett, Joseph Atherton

**Affiliations:** ^1^ Centre for Host-Microbiome Interactions, Faculty of Dental, Oral and Craniofacial Sciences, King’s College London, London, United Kingdom; ^2^ Randall Centre for Cell and Molecular Biophysics, King’s College London, London, United Kingdom

**Keywords:** cryo-EM, ssNMR, filament, fibre, microtubule, pilus

## Abstract

Historically proteins that form highly polymeric and filamentous assemblies have been notoriously difficult to study using high resolution structural techniques. This has been due to several factors that include structural heterogeneity, their large molecular mass, and available yields. However, over the past decade we are now seeing a major shift towards atomic resolution insight and the study of more complex heterogenous samples and *in situ*/*ex vivo* examination of multi-subunit complexes. Although supported by developments in solid state nuclear magnetic resonance spectroscopy (ssNMR) and computational approaches, this has primarily been due to advances in cryogenic electron microscopy (cryo-EM). The study of eukaryotic microtubules and bacterial pili are good examples, and in this review, we will give an overview of the technical innovations that have enabled this transition and highlight the advancements that have been made for these two systems. Looking to the future we will also describe systems that remain difficult to study and where further technical breakthroughs are required.

## Introduction

In the 1930s-1950s, X-ray fibre diffraction studies of filamentous proteins by pioneers such as William Astbury, Francis Crick and Linus Pauling, laid the foundation for modern structural biology (Astbury and Street, 1932; Pauling and Corey, 1951a; Pauling and Corey, 1951b; Crick, 1952). These early studies provided new insights into the structural properties of fibrous substances such as keratin and collagen, however, they could only offer global information. With the advent of single crystal X-ray diffraction (Kendrew et al., 1958), from the 1960s structural studies of soluble proteins became the principal focus due to the delivery of atomic/subatomic resolutions. In the 1980s the first soluble protein was determined by solution state nuclear magnetic resonance (NMR) spectroscopy (Williamson et al., 1985) and with the development of recombinant protein expression systems (Itakura et al., 1977; Smith et al., 1983; Cregg et al., 1993), by the mid-1990s there became a clear exponential rise in soluble/globular macromolecular structures being deposited in the protein data bank (Berman et al., 2000). Likewise, new methods for isolating and reconstituting membrane proteins has led to significant numbers of these structures (Vinothkumar and Henderson, 2010), primarily elucidated by crystallographic methods, being deposited in the PDB since the 2000s.

**FIGURE 1:**
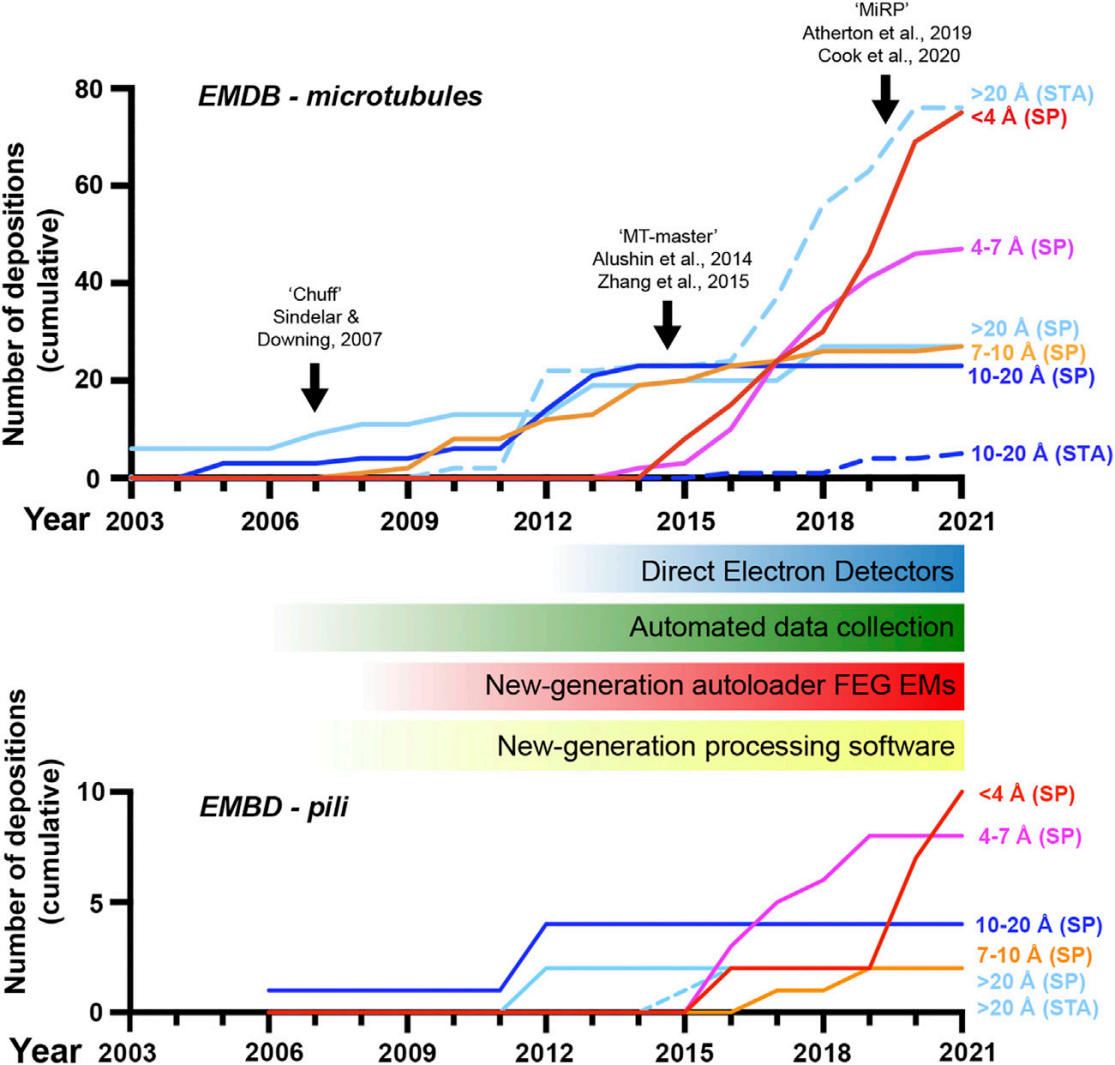
A revolution in cryo-electron microscopy of MTs and pili. Graph showing the cumulative number of electron microscopy database (EMDB) depositions over time at different resolution levels as indicated; SP, single-particle cryo-EM, STA, sub-tomogram averaging. The emergence of new general hardware and software behind the revolution in cryo-EM is shown below the MT timeline, along with black arrows indicating the introduction of several image-processing pipelines for pseudo-helical single MTs in the top graph. Included data was based on the query title:MT AND status:REL or title:pilus AND status:REL at www.emdatasource.org.

Using solution NMR and X-ray crystallography we have gained significant understanding of filamentous systems through studying their lower-order subunits, however, due to technical limitations of these techniques, there has been a lack in our understanding of how these components interact and how this relates to their function. For example, there is a requirement for sample homogeneity (purity and molecular mass), high amounts of material, and for crystallography the sample must form highly ordered and relatively large crystals (generally >20–50 μm^3^). Early advancements in the determination of high-resolution filamentous protein structures include the crystal structure of a synthetic peptide based collagen-like fragment with a defined length, published in 1994 (Bella et al., 1994). In the late 1990s the first proteinaceous structures were determined using solid state NMR (ssNMR) spectroscopy: the helical antibiotic peptide gramicidin, integrated within a lipid bilayer (Ketchem et al., 1996), and a peptide-based pentameric transmembrane helical bundle of the acetyl choline receptor (Opella et al., 1999). This ushered in a new direction for studying highly polymeric solids and by 2003 ssNMR had been successfully implemented to determine the structure of the fd filamentous bacteriophage particle coat protein (Zeri et al., 2003). Since then, the development of smaller magic angle spinning (MAS) sample rotors that spin at higher frequencies and require less sample, advances in isotopic labelling and partial deuteration of samples, and general enhancements of signal sensitivity (Ashbrook and Hodgkinson, 2018), has led to ∼140 ssNMR-derived models being deposited in the PDB (Berman et al., 2000). Of these, ∼20% are of filamentous proteins. Furthermore, in the last 10 years, hybrid approaches incorporating ssNMR, Rosetta-based *in silico* modelling and/or electron microscopy has provided atomic structures for the bacterial type III secretion system needle (Loquet et al., 2012; Demers et al., 2014), the M13 bacteriophage capsid (Morag et al., 2015) and Aβ amyloid fibrils (Sgourakis et al., 2015). However, over the past decade, cryo-electron microscopy (cryo-EM) has transformed all aspects of structural biology and has become the primary driving force in providing major advancements towards routine structure determination of filamentous protein assemblies (Figure 1).

### The Cryo-EM Revolution

Pre-revolution, cryo-EM produced near-atomic resolutions only in ideal sample cases; usually large macromolecules with high symmetry such as particular viral capsids. In additional preferable cases, sub-nanometer resolutions allowing secondary structure visualisation were possible, but for most targets, particularly those of low symmetry and/or small size (<200 kDa), cryo-EM was most-often limited to nm resolutions. Now, cryo-EM can routinely produce near-atomic resolution structures of even asymmetrical macromolecules of small size (currently down to around ∼50 kDa in ideal cases without a scaffold (Fan et al., 2019; Herzik et al., 2019)), with the first true atomic resolutions being reached in the last few years (Nakane et al., 2020; Yip et al., 2020). Furthermore, a significant amount of sample heterogeneity can also now be tolerated and even utilised, with snapshots of different conformational states and multimeric arrangements revealing the dynamics of macromolecular machines. Whilst remaining more challenging than single-particle cryo-EM, in the last 5 years or so cryo-electron tomography (cryo-ET) with sub-tomogram averaging has become more capable of achieving sub-nanometer resolutions *in situ* and near-atomic resolutions with purified macromolecular preparations (Schur, 2019; Turk and Baumeister, 2020; Pyle and Zanetti, 2021).

Core to cryo-EM’s transformation has been the introduction of direct electron detectors to replace charge-coupled devices and film, improving how faithfully transmitted electrons are recorded and allowing correction of global sample drift and local beam-induced motion (Li et al., 2013; Kuhlbrandt, 2014). Improvements to electron microscope hardware (including electron sources, stability, and energy filtration) and software (particularly increased automation in data collection) but also data processing hardware (increased computing power and improved storage) and software (in particular new Bayesian and artificial intelligence-based approaches) have also made significant contributions. The bottleneck to high-resolution cryo-EM is most often now the sample itself or the way it behaves during vitrification, however, innovation in sample preparation and data collection techniques are addressing these challenges.

In this review, we provide several examples of filamentous systems and show how our understanding has developed over the past decade due to these advances in structural biology techniques. We first discuss advancements in our understanding of microtubules (MTs) and MT complexes and then describe bacterial pili, primarily involved in adhesion. Looking to the future, we also highlight aspects that remain difficult to study and suggest where further advancements may be made.

### Microtubules and Associated Proteins

MTs are tubular polymers of around 25 nm diameter built from longitudinally and laterally associated αβ-tubulin heterodimers and are a key component of the eukaryotic cytoskeleton. MTs display dynamic instability, in that they switch between polymerisation and depolymerisation phases modulated by β-tubulin’s GTPase activity, post-translational modifications and the interaction of microtubule-binding proteins. MT dynamics generate forces vital to cell division, serve as intracellular signalling platforms and provide the tracks for intracellular transport and force generation by kinesin and dynein family motor proteins (Vale, 2003; Goodson and Jonasson, 2018). While MTs can form into a number of different architectures built from 10–16 protofilaments, 3-start 13-protofilament pseudo-helices are the most commonly observed in nature (Tilney et al., 1973; Pierson et al., 1978; Wade et al., 1990). Pseudo-helical MTs are those with a discontinuity in the helical lattice known as the seam that has heterotypic (α to β-tubulin) rather than homotypic (α to α/β to β) lateral interactions between tubulin dimers.

### High-Resolution Studies of MTs Using Cryo-EM

MTs were amongst the first targets to be studied by cryo-EM (Mandelkow and Mandelkow, 1985) following its development (Lepault et al., 1983; McDowall et al., 1983). Cryo-EM at this early stage already provided key advantages over EM with heavy metal stains, enabled by rapid sample vitrification in near-native conditions, yet was chiefly limited to theoretical extrapolation of 3D MT lattice architectures *via* analysis of real and reciprocal space patterns from 2D projections (Chretien and Wade, 1991). In the late 90s and early 2000s, 3D reconstructions of MTs with or without associated motor proteins *via* helical and pseudo-tomographic back-projection methods were limited to nanometre resolutions (e.g. (Arnal et al., 1996; Hoenger et al., 1998; Kikkawa et al., 2000; Kikkawa et al., 2001; Metoz et al., 1997; Nogales et al., 1999; Sosa et al., 1997a; Sosa et al., 1997b)) (Figure 1). This work gave information on the MT polymer not available from emerging crystallographic structures of αβ-tubulin or protofilament subunits (Nogales et al., 1995; Nogales et al., 1998; Lowe et al., 2001). An important and lasting shift was treating MT segments as single-particles in reference-matching approaches (Li et al., 2002). This, often combined with refinement of helical symmetrisation parameters during iterative rounds of reference-based alignment (Egelman, 2000), helped move some MT reconstructions into the sub-nanometre range allowing secondary structure identifications (Bodey et al., 2009; Sui and Downing, 2010; Alushin et al., 2012) (Figure 1).

**FIGURE 2:**
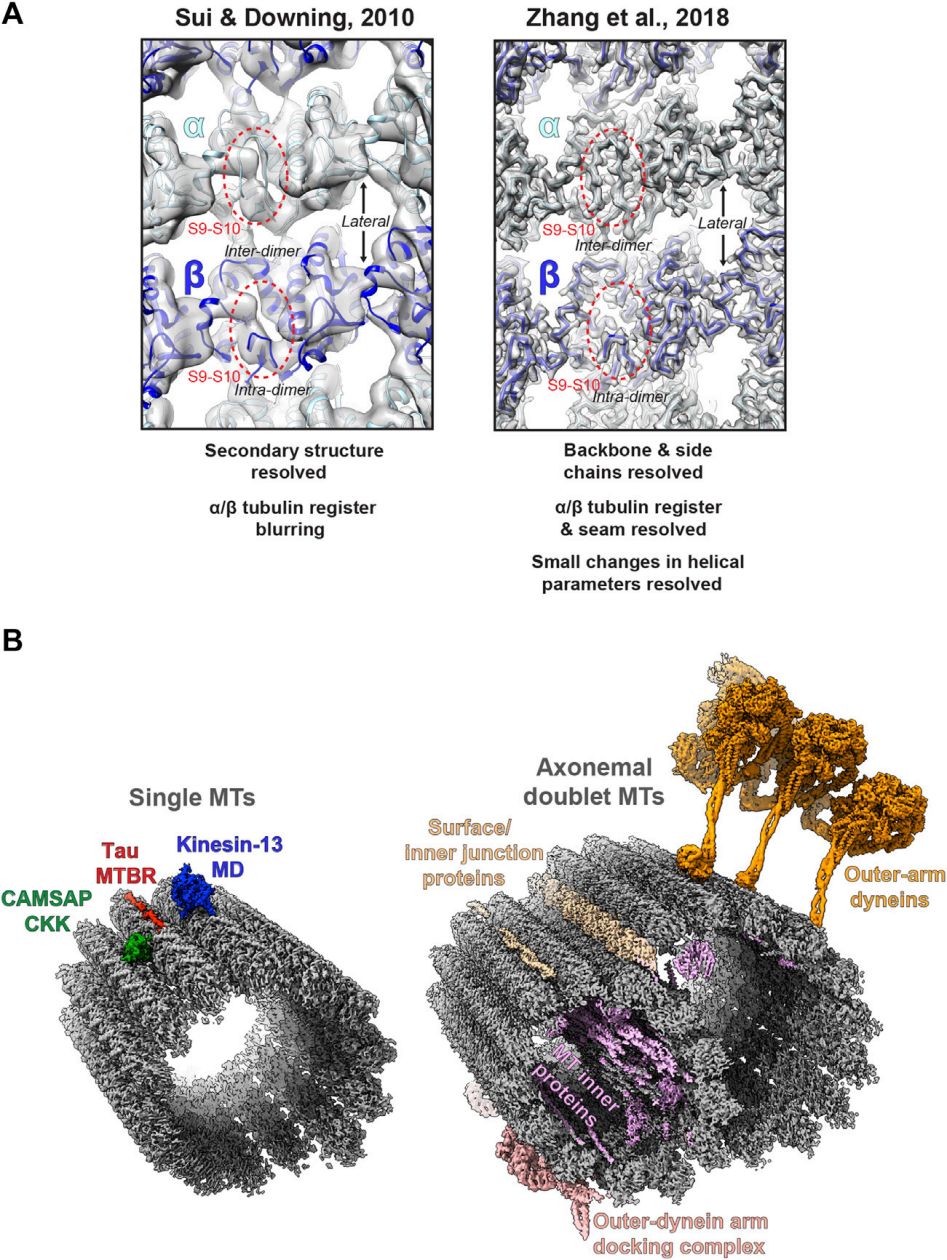
Cryo-electron microscopy of MTs **(A)** Images of the lumenal face of undecorated MTs, centred on an inter-dimer interface, but also showing intra-dimer interfaces and lateral interfaces between protofilaments. Grey density is shown for reconstructions of the MT alone; top, EMDB:5193 at ∼8 Å resolution (Sui and Downing, 2010); and bottom; EMDB:7973 at 3.1 Å resolution (Zhang et al., 2018), with the atomic model for the undecorated GMPCPP MT PDB:6dpu (Zhang et al., 2018) fitted into each reconstruction (α-tubulin light blue, β-tubulin dark blue). Failure to resolve differences between α and β-tubulin is a symptom of the earlier study (top), but not the more recent study (bottom), as illustrated by poor (top) or good (top) density differentiation between α and β-tubulin’s S9-S10 loop (within red dashed oval) **(B)** Left; exemplar cryo-EM structures from single MTs. Coloured cryo-EM densities for MT-binding proteins are shown as indicated on the single MT alone cryo-EM density map EMDB:7973 (Zhang et al., 2018) coloured grey; CAMSAP1 CKK domain (Atherton et al., 2019), MT-binding repeat (MTBR) of Tau (Kellogg et al., 2018) and the motor domain (MD) of kinesin-13 (Benoit et al., 2018). Right; exemplar cryo-EM structures from axonemal doublet MTs. Coloured cryo-EM density for MT-binding proteins are shown as indicated on the bovine tracheal cilia doublet-MT cryo-EM density map EMDB:24664 (Gui et al., 2021); bovine tracheal cilia MT inner proteins (MIPs), surface and innter junction proteins and outer-dynein arm docking complex EMDB:24664 (Gui et al., 2021) and outer-arm dynein from *Tetrahymena thermophila* EMDB:22677 (Rao et al., 2021).

A major challenge is differentiating between highly similar α and β-tubulin monomers during MT image processing, resulting in significant blurring of α and β-tubulin and a failure to resolve the seam in more physiologically relevant pseudo-symmetric MTs (Figure 2A). This issue was particularly prominent in studies of MTs alone, while the presence of MT-bound proteins demarcating tubulin dimers would act as fiducials during processing, alleviating the severity of the artefact, particularly for pseudo-symmetric MT architectures. Combining statistical methods for seam-finding with pseudo-symmetrical averaging approaches efficiently identified α and β-tubulin register and seam-location with MT-binding proteins acting as fiducials (Sindelar and Downing, 2007). Nevertheless, CCD and film-derived reconstructions of pseudo-helical MTs and associated proteins could not break the ∼4.5 Å resolution barrier required for visualisation of the peptide backbone and side chains (Alushin et al., 2014; Atherton et al., 2014; Fourniol et al., 2010; Maurer et al., 2012; Redwine et al., 2012; Sindelar and Downing, 2010). The introduction of direct electron detectors was central to MT reconstructions achieving near-atomic resolutions (<4.5 Å) (Figure 1). Ground-breaking work from the Nogales group, combining direct electron detector data with refined pseudo-helical processing methods achieved resolutions around 3.4 Å, allowing the authors to propose that small local nucleotide-dependent conformational changes leading to global changes in lattice compaction and twist govern dynamic instability (Zhang et al., 2015; Zhang and Nogales, 2015). This and later work to near-atomic resolutions continued to use MT binding proteins as fiducials for differentiation of α and β-tubulin in processing when working on tubulin isoforms or nucleotide or drug-induced conformational changes in the MT lattice; (Howes et al., 2018; Kellogg et al., 2017; Manka and Moores, 2018; Vemu et al., 2017; Vemu et al., 2016). However, this need for fiducials was overcome with additional image processing techniques, when near-atomic structural determination of pseudo-symmetric MTs without binding proteins in different nucleotide states was demonstrated (Zhang et al., 2018) (Figure 2A). Nevertheless, in many cases the MT-binding protein itself was the subject of structural interest, with notable examples solved near-atomic resolutions including the MT-binding regions of disease-related proteins tau (Kellogg et al., 2018) and doublecortin (Manka and Moores, 2020), minus-end binding CAMSAP (Atherton et al., 2019), MT-nucleator TPX2 (Zhang et al., 2017) and the MT-depolymerising kinesin-13 (Benoit et al., 2018) (Figure 2A).

In recent years, cryo-EM of MTs and binding partners has continued to develop (Figure 1). Near-atomic resolutions are being more readily achieved with accelerated automated and semi-automated data collection approaches (Tan et al., 2016) being adopted, allowing large amounts of data to be collected. Pipelines and methods for image processing of pseudo-symmetric MTs with or without MT-binding protein fiducials in the popular GUI-based program RELION have been introduced (Adib et al., 2019; Lacey et al., 2019; Cook et al., 2020). One challenge has been MT surface-binding proteins commonly being resolved at lower resolutions than the MT scaffold because heterogeneity in MT shape is generally amplified away from the centre of reconstructions, but also because of sample-dependent sub-stoichiometric occupancies and flexibility in the binder. In response to these challenges, new techniques have been developed to essentially subdivide MTs and bound proteins into sub-regions during processing, for example by using symmetry expansion and/or focused classification and refinement of individual MT subunits or binder sites to improve their quality (Liu et al., 2017; Debs et al., 2020; Cook et al., 2021). A recent stand-out study used focused classification methods to resolve MT inner proteins (MIPs) in the lumen of MTs extracted from *Toxoplasma gondii* to near-atomic resolution (Wang et al., 2021).

Although generally lagging-behind single-particle cryo-EM in resolution (Figure 1), cryo-ET can reveal 3D information on structurally heterogenous MT regions, such as lattice breaks and MT ends (Guesdon et al., 2016; Atherton et al., 2017; Gudimchuk et al., 2020). Furthermore, cryo-ET can be used to study MT architecture and organisation either *in situ* or in isolated *ex-vivo* preparations, where crowded overlapping environments render single-particle cryo-EM unsuitable (Atherton et al., 2018; McIntosh et al., 2018; Chakraborty et al., 2020). In particular cases where there are suitable repeating MT-associated sub-structures, sub-tomogram averaging (STA) can be employed to yield isotropic 3D reconstructions and improve resolution. For example, recently cryo-ET has resolved *ex-vivo* cytoplasmic dynein-dynactin transport teams on MTs (Chowdhury et al., 2015; Grotjahn et al., 2018), Parkinson’s disease related LRRK2-decorated MTs in cells (Watanabe et al., 2020), EB-decorated singlet MTs inside primary cilia (Kiesel et al., 2020) and revealed MT intra-lumenal F-actin in kinesore-induced cell projections (Paul et al., 2020).

Finally, alongside work on single MTs and their binding proteins, there has been a recent flurry of exciting cryo-ET/STA (Jordan et al., 2018; Zabeo et al., 2018; Owa et al., 2019; Greenan et al., 2020) and single-particle cryo-EM (Ma et al., 2019; Khalifa et al., 2020; Gui et al., 2021; Rao et al., 2021; Walton et al., 2021) work on the axonemal MT doublet structures of primary and motile cilia (Figure 2B). These studies have utilised both intact *in situ* and reduced membranated or de-membranated *ex vivo* preparations and have revealed a wealth of information on gross cilia architecture and the organisation, identities, and structure of axonemal MIPs, dynein complexes and intraflagellar transport trains (IFTs).

### Studying Dynamic Interactions of Microtubules and Associated Proteins

Prior to cryo-EM’s revolution, ssNMR had provided a method of obtaining high-resolution information on drug-binding to MTs (Kumar et al., 2010). Nowadays, cryo-EM has become the tool of choice for studying the rigid interactions between MTs and their binding partners including small-molecules. However, cryo-EM struggles to resolve significant dynamics and flexible interactions due to the requirement for particle averaging and therefore ssNMR provides an ideal high-resolution method for studying the nature of these more dynamic interactions. Recent studies with labelled MT-binding domains of CAMSAPs, dynactin CAP-Gly, tau and plant companion of cellulose synthase 1 (CC1) have revealed atomic-level information on the dynamicity of their MT interfaces (Yan et al., 2015; Kadavath et al., 2018; Atherton et al., 2019; Kesten et al., 2019).

An exciting new development has been the purification of suitable amounts of isotopically labelled tubulin to produce MTs suitable for NMR studies, allowing labelled MTs and MT-binding proteins to be studied in parallel (Luo et al., 2021). In particular, this has enabled the study of the flexible and isoform-variable C-terminal tails of α and β tubulin involved in a plethora of interactions with MT-binding proteins within intact MTs (Janke, 2014; Roll-Mecak, 2020). This technical advance has now revealed the dynamic involvement of these C-terminal tails in CAMSAP-CKK domain and MAP7 MT binding at both slow and fast timescales (Luo et al., 2020) and has opened the door to further studies with a range of binding proteins.

### Adhesive Bacterial Pili

The extracellular surfaces of bacteria are decorated with hair-like projections called pili or fimbriae, that are composed of smaller pilin subunits (Thanassi et al., 2012; Hospenthal et al., 2017a). Different types of pili range in their length and thickness and often have dedicated export and assembly systems that allow them to form on the bacterial surface. These include type IV-like and type V, chaperone-usher, amyloid-based, conjugative, type IV secretion, and sortase-mediated pili (Lukaszczyk et al., 2019). These pili have diverse functions including interacting with host cells during colonisation, promoting bacterial aggregation in biofilm formation, motility, conjugation, and secretion of proteins (Garnett and Matthews, 2012; Thanassi et al., 2012; Arutyunov and Frost, 2013; Berry and Pelicic, 2015). As such, these filamentous structures are often major virulence factors that drive the establishment of bacterial infection and the progression of disease. A decade ago, our primary understanding of pilus architectures was through crystallographic and solution NMR studies of monomeric pilin domains and the modelling of intact pili using low resolution (>10 Å) negative-stain and cryo-EM data (Craig et al., 2006; Salih et al., 2008; Garnett et al., 2012; Galkin et al., 2013). However, in the past 5 years ∼15 intact pilus structures have now been deposited in the PDB derived using cryo-EM data at near near-atomic resolutions (<4.5 Å) (Costa et al., 2016; Hospenthal et al., 2016; Hospenthal et al., 2017b; Spaulding et al., 2018; Filman et al., 2019; Wang et al., 2019; Zheng et al., 2019; Neuhaus et al., 2020; Shibata et al., 2020; Zheng et al., 2020; Gu et al., 2021; Pradhan et al., 2021). There has been a clear advancement in our ability to resolve high-resolution features of intact bacterial pili and this is well aligned with the introduction of direct electron detectors and processing techniques developed for filamentous helices (Figure 1). Here we will now discuss progress made in our understanding of two major classes of adhesive pili.

### Type IV-like Pili

**FIGURE 3:**
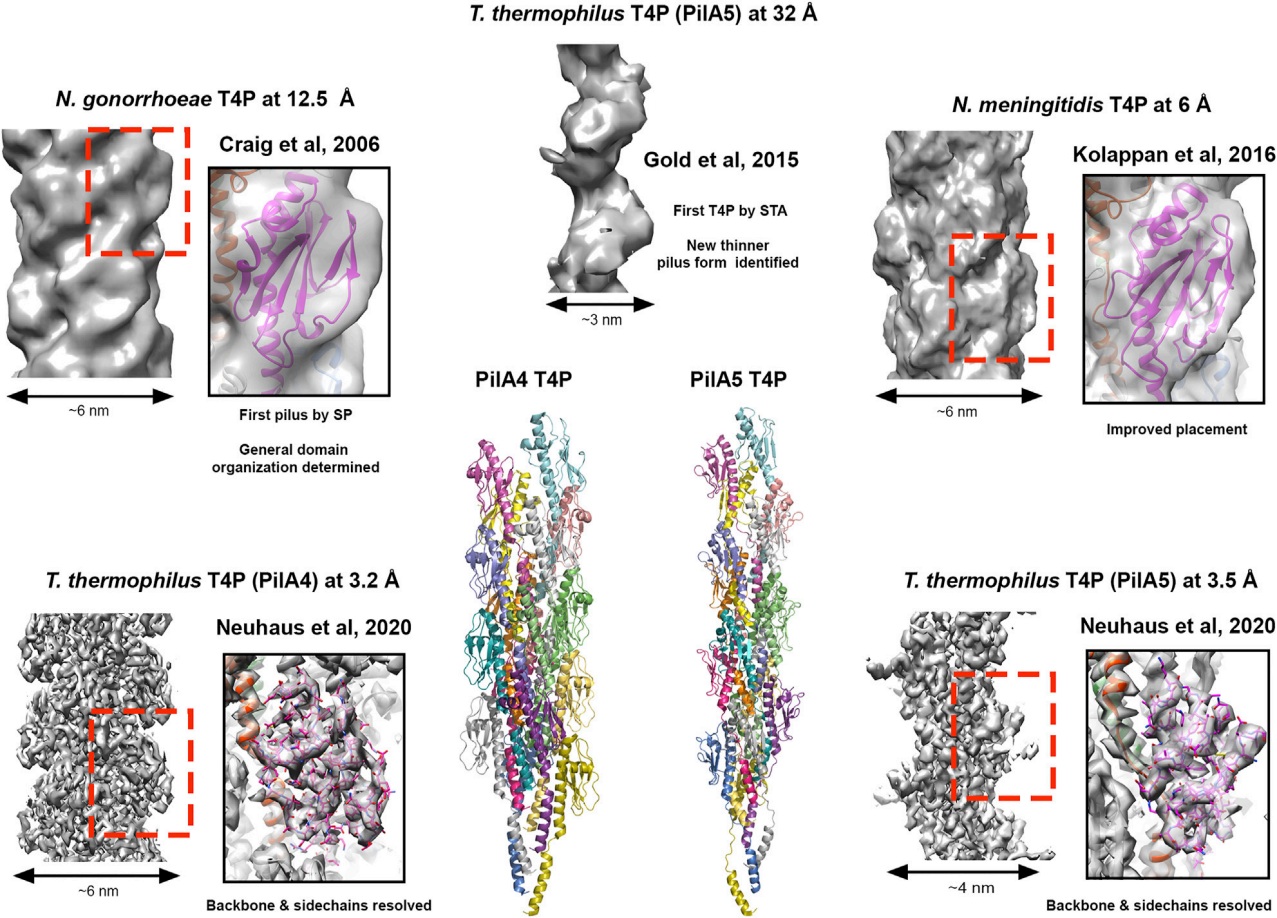
Cryo-electron microscopy of type IV pili. Images of T4P models derived from different resolutions of SP data STA data. Structures providing resolution breakthroughs are shown as example. SP Cryo-EM density and models of “thick” pili (*N. meningitidis* T4P at 12.5 Å (EMDB 1236; PDBID 2hil) (Craig et al., 2006), *T. gonorrhoeae* T4P at 6.0 Å (EMDB 8287; PDBID 5kua) (Kolappan et al., 2016) and the *T. thermophilus* PilA4 T4P at 3.2 Å (EMDB 10647; PDBID 6xxd) (Neuhaus et al., 2020)) and a thin “pilus” (*T. thermophilus* PilA5 T4P at 3.5 Å (EMDB 10648; PDBID 6xxe) (Neuhaus et al., 2020)) are shown. Cryo-EM density for the thinner *T. thermophilus* PilA5 T4P derived by STA at 32 Å is also presented (EMDB 3024) (Gold et al., 2015). Cartoon representation of the *T. thermophilus* PilA4 and PilA5 are also given as the first Cryo-EM backbone/sidechain resolved T4P structures (Neuhaus et al., 2020).

The first pilin structure was published in 1995 by John Tainer’s group; the crystal structure of an intact type IV pilus (T4P) major subunit, PilE, from *Neisseria gonorrhoeae* (Parge et al., 1995). In *N. gonorrhea* this pilus is the only known virulence factor required for infection (Kellogg et al., 1963; Swanson et al., 1987) and is important for binding host cells, other bacteria and delivering antigenic variation (Quillin and Seifert, 2018). Furthermore, the ability of the T4P to both extend and retract, coupled with its adhesive properties, provide bacteria with twitching motility and the ability to conjugate genomic material (Pelicic, 2008). The structure of PilE indicated that a long N-terminal helix could mediate pilus assembly, and the globular C-terminal region may decorate the pilus and provide specific function. In 2006 this was realised using 12.5 Å cryo-EM data to model the intact pilus (Craig et al., 2006) which displayed a tight helical packing of N-terminal helices and with a width of ∼6 nm (Figure 3). Then in 2015, cryo-ET was used to resolve the overall features of the PilA5 T4P isolated from Thermus thermophilus at 32 Å by STA (Figure 3). Strikingly, this pilus displayed a pronounced groove running along the fibre length and was much thinner at ∼3 nm (Gold et al., 2015). Over the past 5 years we have seen significant improvements in resolution first with the *Neisseria meningitidis* T4P using cryo-EM maps reconstructed at 6 Å (Kolappan et al., 2016) and then several other T4P models published guided by sub-nanometre resolution data from *Escherichia coli*, *N. gonorrhoeae* and *Pseudomonas aeruginosa* PAK (Bardiaux et al., 2019; Wang et al., 2017) (Figure 3). All these pili formed structures with widths of ∼5–6 nm. However, in the last year Vicki Gold’s group has now broken the ∼4.5 Å barrier, with cryo-EM maps for the Thermus thermophilus PilA4 and PilA5 pili at 3.2 Å and 3.5 Å, respectively, allowing backbone and sidechain details to be resolved as well as sites of glycosylation (Neuhaus et al., 2020) (Figure 3). Similar to the *Neisseria*-like pili, the PilA4 pilus has a width of ∼6 nm, while PilA5 is in line with the previous cryo-ET study with a width of ∼4 nm. This study has provided a clear atomic rational for the differences in overall appearance of these two classes of pili, which likely reflects their specific functions in natural transformation and twitching motility, respectively, and is based on unique inter-subunit interactions, helical parameters, and surface charge.

Related to the T4P, pseudo-pili have similar structures but different functions. For example, components that form the T4P system and are essential for pilus assembly are related to the type II secretion system (T2SS) which transport substrates and effectors, many of which are virulence factors, from the Gram-negative bacterial periplasm into the extracellular space (Gu et al., 2017). While the T4P system utilise ATPases that can both drive the formation and retraction of the pilus, the T2SS only contains expresses the extension ATPase, which it uses to push cargo across the bacterial outer membrane *via* polymerisation of the pilus *via* a syringe-like mechanism. In 2017 the structure of the *Klebsiella oxytoca* T2SS pseudo-pilus, PulG, was resolved using solution NMR and cryo-EM maps at 5.0 Å resolution, and this study has revealed that this pilus is stabilised by calcium ions and disassembles in their absence (Lopez-Castilla et al., 2017; Naskar et al., 2021). *Geobacter sulfurreducens* is a Gram-negative bacterium that uses surface nanowires for extracellular electron transfer. A recent cryo-EM structure of the copolymerised PilA-N/C pseudo-pilus at 3.8 Å now indicates that like the T2SS, this structure is used to push nanowires out of the bacterium (Gu et al., 2021). Furthermore, cryo-EM structures of the OmcS nanowire at 3.7 Å and 3.4 Å has uncovered a new pilus type formed through the polymerisation of OmcS hexaheme cytochromes, with hemes packed within 3.5–6.0 Å of each other to allow electron transport (Filman et al., 2019; Wang et al., 2019).

### Donor-Strand Exchanged Pili

The chaperone-usher (CU) pilus assembly pathway is another well characterised system in Gram-negative bacteria with the first structure, the uropathogenic *E. coli* (UPEC) type 1 pilus minor FimC-FimH chaperone-adhesin complex, published at the end of the 1990s by Stefan Knight’s group. In CU systems, pilin domains consist of an incomplete Ig-like fold which lack the C-terminal strand, forming an acceptor groove, but have an additional unstructured extension at their N-terminus (Hospenthal et al., 2017a). Polymerisation proceeds through this N-terminal extension packing along the acceptor groove of an adjacent pilin subunit, which then stabilises and completes the Ig-like fold; a process called donor strand exchange. CU pili are highly variable but many consist of a major pilin subunit that makes up the majority of the fibre and then a minor pilin subunit (one or a few) at the tip, which is often an adhesin that binds carbohydrates or other receptors on the surface of host cells (e.g. *E. coli* type 1, P and common pili) (Garnett et al., 2012; Hospenthal et al., 2017a). However, in other CU systems, the major pilin domains that form the fibre shaft instead act as adhesive elements while the minor tip domain functions as an invasin that mediates invasion of host cells (e.g. *E. coli* AAF pili) (Berry et al., 2014); other arrangements also exist. Additionally, some CU pili are relatively thin (∼2–3 nm) and exist with an extended ‘beads on a string’ architecture that are relatively dynamic, with others being much thicker (∼10 nm) and forming rigid and more compacted helical arrangements.

A decade ago our understanding of CU pilus structures was primarily driven by crystallographic and NMR studies of generally monomeric or small engineered tandem subunits, however, again due to advances in cryo-EM we are now able to appreciate the functions of pilus packing through near-atomic resolution insights (Hospenthal et al., 2016; Hospenthal et al., 2017b; Spaulding et al., 2018; Zheng et al., 2019). In 2013 Lisa Craig’s group determined the global features of the enterotoxigenic *E. coli* (ETEC) CS1 pilus at 20 Å resolution by cryo-EM and the major CS1 pilin subunit CooA by crystallography at 1.6 Å resolution (Galkin et al., 2013). Modelling the CooA subunits into different cryo-EM maps revealed that pilins could adopt multiple orientations and structural states, resulting in different pilin packing and a dynamic pilus structure. They proposed that CS1 and other thicker class of pili may stretch in response to shear forces that they experience during colonisation. In 2015 Adam Lange’s group combined solution state NMR, ssNMR and scanning transmission electron microscopy (STEM) and determined the first atomic model of an intact UPEC type 1 pilus (Habenstein et al., 2015) while the following year, Gabriel Waksman’s group published the first near-atomic resolution structure of an intact UPEC P-pilus by cryo-EM at 3.8 Å (Hospenthal et al., 2016). This structure along with a subsequent structure of the UPEC type 1 pilus by cryo-EM at 4.2 Å was able to explain precisely how changing inter-subunit interactions within these pili can mediate spring-like properties (Hospenthal et al., 2017b; Spaulding et al., 2018). Another recent study of the ETEC CFA/I pilus by cryo-EM at 4 Å has again shown that the helical quaternary structure of the pilus is influenced by shear forces and this is likely a common function of wound CU pili (Miller et al., 2006; Zheng et al., 2019).

Within the past 5 years a new type of adhesive fibre has also been discovered in Bacteroidales, named the type V pilus (T5P) (Xu et al., 2016). These are composed of an anchor, a stalk, an adapter and a tip pilin (Hospenthal et al., 2017a). Last year the first structure of an intact T5P was determined by cryo-EM at 3.6 Å, which consisted of the polymerised FimA pilin stalk subunit from the bacterium *Porphromonas gingivalis* (Shibata et al., 2020). This has revealed not just a new pilus architecture but also a new mode of pilus formation. Unlike the CU pathway, T5P pilin domains contain a C-terminal extended region and when they are exported to the bacterial surface, the N-terminal strand is cleaved by the protease RgpB and released. This forms an acceptor groove that can then accept a C-terminal extension *via* strand exchange with an adjacent pilin subunit.

## Conclusions and Perspectives

As has been discussed, structural biology of MTs and their binding proteins has advanced dramatically over the last decade, yet a number of samples and technical goals remain challenging, representing frontiers in the field. In single-particle cryo-EM of single MTs, an intriguing question is whether atomic resolutions are achievable. MTs include some intrinsic flexibility and heterogeneity, including bending and in some cases lattice breaks and even multiple seams (Cross, 2019; Debs et al., 2020). Future sample preparation, data collection and processing methods will have to reduce these variances, whilst essentially eliminating blurring of α and β-tubulin register (particularly challenging in the absence of fiducial binding proteins) in order to reach atomic resolutions. Resolving MT binding proteins with partial occupancy on the MT, or those adopting a mix of conformers remains difficult, although processing strategies including symmetry expansion, focused classifications and refinements and signal subtraction are proving fruitful (Liu et al., 2017; Debs et al., 2020; Cook et al., 2021). In some cases, rather than using the standard heterogenous tubulin purified from the brains of livestock, more homogenous sources such as purified single-isoform tubulins or tubulin from particular cell types can help improve resolve regions of tubulin isoform heterogeneity, such as the C-terminal tails and their interactions (Atherton et al., 2019; Li et al., 2020). Additionally, ssNMR is becoming a useful tool for particular cases where flexible interactions between MT-binding proteins and the MT are to be investigated.

As discussed, exciting recent data has also emerged from studies of cilia MTs that demonstrates the ability of cryo-EM to identify macromolecules *de novo* within *in situ* or *ex vivo* preparations (Gui et al., 2021; Kiesel et al., 2020; Wang et al., 2021). The ability to faithfully localise and identify macromolecules on MTs *in situ* is likely to be a prominent target in the future direction of the field. With the advent of a revolution in macromolecular structure prediction (Jumper et al., 2021) combined with expanding dataset sizes and increasing sub-tomogram averaging resolutions due to steady improvements in cryo-ET sample preparation (FIB), data-collection and image processing (Figure 1) (Turk and Baumeister, 2020; Hylton and Swulius, 2021; Pyle and Zanetti, 2021), this goal is looking more and more plausible.

On the back of cryo-EM method developments, primarily through the study of other filamentous systems such as MTs, there has also been a substantial increase in our understanding of bacterial pilus structures in recent years. This is highlighted by work shown here on type IV-like pili, CU pili and the newly characterised T5P, however, we still lack high-resolution insight of other types of pili, such as those formed from amyloids (Van Gerven et al., 2018), and lack an understanding of how minor pilin components interact within intact pilus assemblies. In many type IV pili, incorporation of minor pilins is required for pilus assembly and/or specific functions (Jacobsen et al., 2020) but it is unclear how these are distributed across the fibre or what effect these minor components have on the local pilus structure. Crystallographic and more recently cryo-EM studies have provided great insight into the atomic mechanisms of pilus biogenesis (Hospenthal et al., 2017a), however, pili often remain tethered to their secretion machinery, and there is a shortage of high-resolution observations in these states. The UPEC type 1 and P pili have become model systems to study CU pilus biogenesis and several groups have now provided snapshots of the initiation of pilus assembly at the outer membrane usher pore and the initial stages of adhesin exit into the extracellular space (Phan et al., 2011; Geibel et al., 2013; Du et al., 2018; Omattage et al., 2018; Du et al., 2021). With cryo-EM now leading the way here, the next leap will likely come once a defined number of major pilin subunits can be incorporated into these systems and then the maturation of the pilus can then be visualized in the context of secretion.

Although cryo-ET has not been used to study the CU pathway, it has been successfully implemented to elucidate the low-resolution features of T4P and T2SS pseudo-pilus assembly devices (Gold et al., 2015; Chang et al., 2016; Ghosal et al., 2019). Work on the T4P systems has revealed pilus structures emanating into the extracellular space (Gold et al., 2015; Chang et al., 2016) and with the expected future increases in cryo-ET resolution, as discussed above, in the future cryo-ET may be able to provide greater detail of how these pili form at the membrane and the role of pilus-specific assembly factors in their biogenesis (Hu et al., 2020). Cryo-EM has had a major impact on the of study filamentous systems over the past decade and it will be interesting to see how EM and its incorporation with other approaches (e.g. *in silico* prediction, solution/ssNMR, crystallography) will be able tackle more dynamic and heterogenous filamentous systems in the future.
